# Role of *Porphyromonas gingivalis*’s peptidylarginine deiminase in multispecies biofilm formation and bacterial adherence to host cells

**DOI:** 10.1080/20002297.2017.1325247

**Published:** 2017-05-30

**Authors:** Ardita Aliko, Marta Kamińska, Brith Bergum, Annelie Hellvard, Roland Jonsson, Piotr Mydel

**Affiliations:** ^a^ Broegelmann Research Laboratory, Department of Clinical Science, University of Bergen, Bergen, Norway

## Abstract

*Porphyromonas gingivalis*’s peptidylarginine deiminase (PPAD) is one of the most unique virulence factors in the pathogenesis of periodontitis, as well as one of the possible links between this chronic inflammatory disease and other disorders, like rheumatoid arthritis. However, it is yet unclear how it is involved in the infection of epithelial cells, therefore the aim of this study was to examine whether PPAD enzyme has an effect on the formation of biofilm by *P. gingivalis* in consortium with four other bacteria species, and the bacterial adhesion and invasion of gingival keratinocytes and their subsequent response. Using PPAD-deficient strains, we have demonstrated that its activity has no effect on *P. gingivalis*’s ability to form a biofilm, nor does it change the species composition in such a formation. Moreover, flow cytometry analysis of the adhesion and invasion of keratinocytes by those bacteria did not reveal any difference depending on PPAD activity, which was further supported by the analysis of transcriptome, as expression of genes involved in the process of internalization of bacteria were not affected. Therefore, we conclude that PPAD activity does not play any role during biofilm formation or *P. gingivalis*’s ability to adhere to and enter host’s cells.

